# Resolution of Confusion Over Compartment Syndrome After Tibial Osteotomy With Continuous Pressure Measurements

**DOI:** 10.7759/cureus.61114

**Published:** 2024-05-26

**Authors:** Abdullah Haidar, Thierry Pauyo, Edward Harvey, Justin Drager

**Affiliations:** 1 Orthopedic Surgery, McGill University, Montreal, CAN

**Keywords:** technology, emergency, sensors, compartment syndrome, trauma, osteotomy, surgery

## Abstract

Compartment syndrome (CS) can occur in a variety of clinical scenarios. Reperfusion injury and tissue swelling are common causes across etiologies. Trauma is recognized as a common cause, but CS is also seen after limb alignment correction for extremities. CS is a difficult diagnosis to make in any scenario. Timely diagnosis is also difficult. Correct diagnosis is inexact, with many false positives and some false negatives being the normal outcome. This case represents a scenario where it was inherently difficult to make the diagnosis. The patient was a young patient with an underlying neurodevelopmental disorder where physical and clinical examination was impossible to accomplish. Any intervention to decrease pain was also difficult and actively refused by the patient and the family. Leaving open wounds after a fasciotomy was also undesirable for wound care and infection. Previous care maps have high false-positive rates or a need for fasciotomy as the treatment arm when diagnosis is uncertain. This usually results in fasciotomy being performed in many legs without CS. These false positives and resultant prophylactic releases are costly because of protracted hospital stay, high rate of deep infection, and decreased operating room availability for other cases. The desirable tool for surgeons would be the one that decreased false positives and false negatives while ensuring diagnosis in a timely fashion for true-positive cases. Technology for monitoring continuous pressure has been shown to aid in diagnosis. In this report, we illustrate the use of a continuous pressure monitoring system in a case of a pediatric patient post-osteotomy of a lower limb presenting with unremitting pain and a difficult clinical examination.

## Introduction

Compartment syndrome (CS) continues to be a limb-threatening complication. The diagnosis of CS still relies on clinical findings of increasing pain, paresthesia, pallor, pulselessness, paralysis, and poikilothermia (also called “the 6P’s”). Arguably, the 6Ps are not good indicators for CS and many may be more appropriate for vascular injury, but they continue to be the clinical benchmark. The 6Ps are not objective diagnostic markers, relying on subjective evaluations that may or may not be reproducible between surgeons and other centers. This case illustrates a scenario where it is almost impossible to rely on clinical signs to provide a diagnosis. The patient presented 36 hours after a tibial tubercle osteotomy with severe pain and an inability to tolerate any physical examination or attempts to enhance the presenting diagnosis. Pressure has been used to aid in diagnosis, but, in the literature, there has been difficulty with objective values that have illustrated lessened reliability on single stick pressure measurement [1]. Early methods and devices used for measuring intracompartmental muscle pressure are well known to have limited accuracy and reliability [1-3]. Fortunately, the rate of CS in tibial osteotomy or leg realignment surgery is reported to be rare [4]. This does not take away the morbidity and risk associated with CS. Monitoring continuous pressure is accurate, decreases time to surgery, and aids in diagnosis [5-7], especially with the new more reliable methods for measurement. The standard treatment of CS still consists of an emergency fasciotomy that immediately reduces the pressure within the affected compartment. It has been shown that the number of fasciotomies vastly outnumbers the incidence of CS in the United States [8]. This would indicate that the current techniques used for the diagnosis or treatment of CS are insufficient and therefore result in unnecessary surgeries. There are attendant complications with all open wounds, and this is no different for fasciotomies. The fasciotomy wound is often left open for several days, which carries a high risk of infection and non-union. In the case of this young patient, the surgeons did not feel that patient care would be optimized if fasciotomies were performed and that wound care would be very difficult. Use of MEMS (micro-electrical machine systems) based pressure monitoring has shown excellent results for extremity injuries [6]. This type of device was implemented as a diagnostic aid in this case to allow diagnosis.

## Case presentation

**Figure 1:**
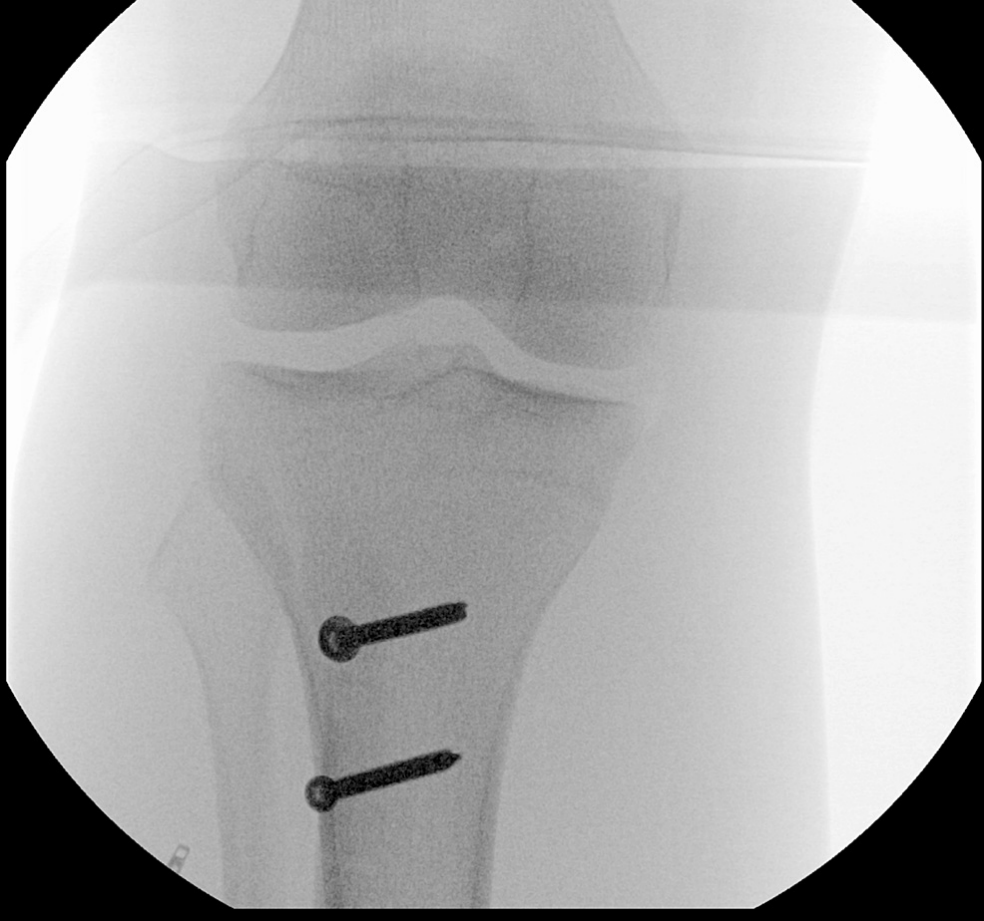
AP radiograph of the operative site at the knee. AP radiograph taken in the operating room shows no displacement of the fixation or breakage of hardware. AP, anteroposterior

**Figure 2:**
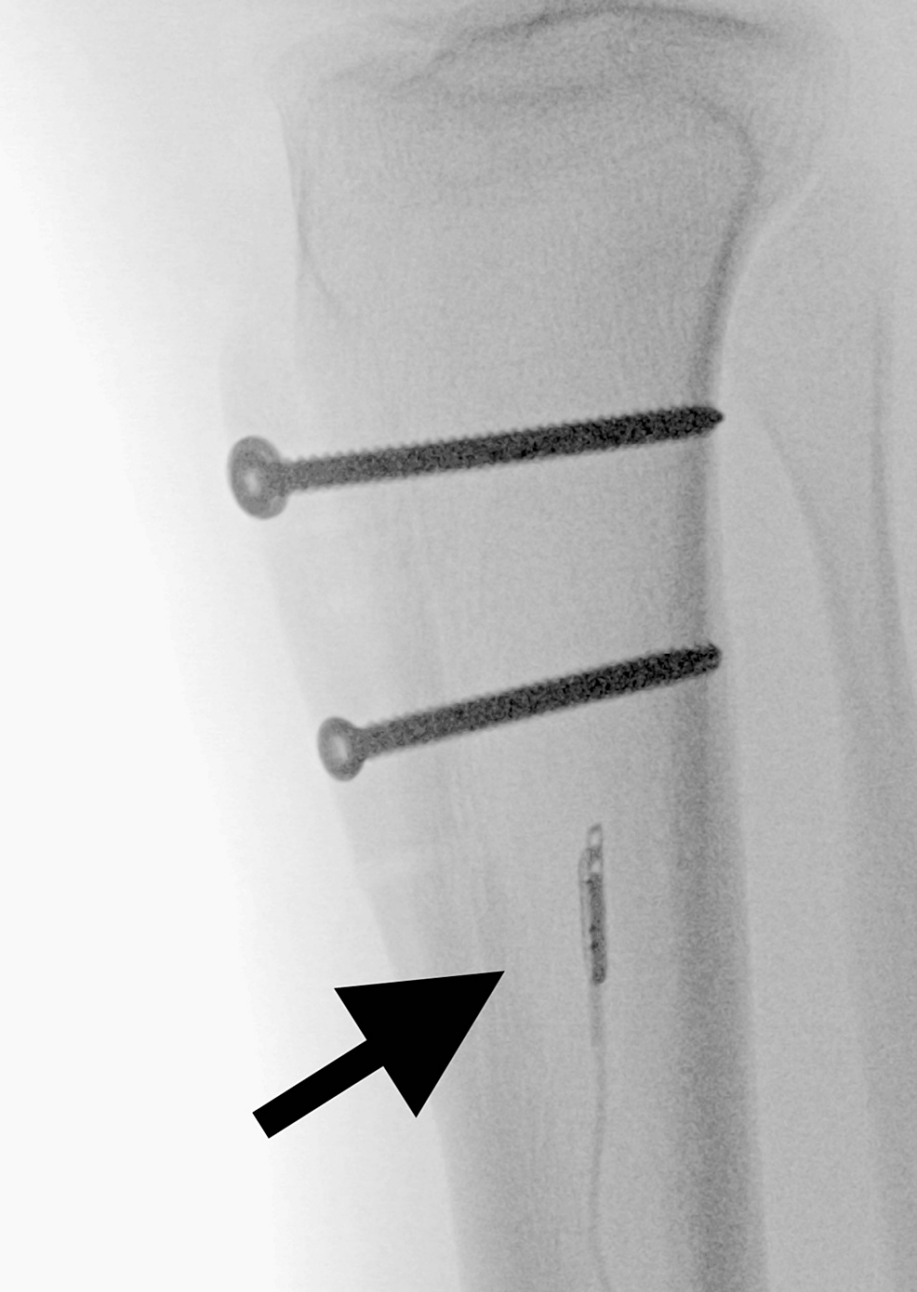
Lateral radiograph of the previous operative site. Lateral radiograph taken in the operating room shows no displacement of the fixation or breakage of hardware. Arrow shows sensor in good place in the muscle belly.

A 13-year-old near skeletally mature female presented with a history of recurrent patellar dislocation. Her past medical history included autism spectrum disorder, anxiety, and obsessive-compulsive disorder. She failed non-surgical treatment, and after a thorough discussion of the pros and cons of surgical management, she underwent medial patellofemoral ligament reconstruction and a tibial tubercle osteotomy to correct this problem (Figures 1, 2).

Surgery was performed by an experienced sports medicine surgeon, and there were no interoperative complications. She was discharged the next day with a continuous peripheral adductor canal nerve block that had been placed in the operating room. She was in a hinged knee brace locked in full extension and was non-weight bearing on crutches. She was taking a regular dose of acetaminophen, ibuprofen, and morphine optimized for her weight by the pain service. The next day at 36 hours after surgery, she began to have increased unremitting pain in her operated leg. She presented to the emergency department.

The wounds were clean and dry with no signs of dehiscence/discharge or infection. She had tenderness globally in the operated knee and leg with mild swelling of her knee and lower leg. Her distal vasculature was intact with dorsalis pedis and posterior tibialis pulses present. All dermatomes were sensory intact but accentuated. Her capillary refill was normal, with no pallor of the foot or toes. There was no obvious venous engorgement. The patient had severe pain with any movement. She was unable to dorsiflex her toes, and passive motion increased the pain globally. Radiographs were not possible at this time due to patient agitation. The patient was afebrile but tachycardic. Laboratory results showed a slightly elevated C-reactive protein with normal white blood cell count. D-dimer result was low.

She had a soft calf, but it was extremely challenging to assess without sedation due to the patient being in pain and combative with any attempt at a thorough physical examination. She was sent for ultrasound to rule out deep venous thrombosis. The radiologist was unable to definitely rule out clotting because the pain was unbearable for the patient in all compartments. The study was abandoned before conclusion. The patient and the family were refusing any attempt at performing another nerve block without complete sedation. Deep palpation showed muscle tightness with diagnosis being either CS or spasm. Overall, her pain and agitation were out of proportion, and CS needed to be ruled out. A heightened reaction to expected postoperative pain and muscle spasm was also possible after assessment of the overall clinical situation, and the morbidity of performing immediate fasciotomies for this patient was also strongly considered.

It was opted to perform continuous intercompartmental pressure monitoring to aid the clinical examination. The patient and family were extremely resistant to the insertion of any device through the skin. A discussion with the family resulted in a care plan that would allow pressure measurements. The patient consented for fasciotomy, but the plan was to induce anesthesia to decrease muscle spasm and allow insertion of the pressure sensor device. It was hoped to avoid the fasciotomy that was indicated by the current clinical scenario.

**Figure 3:**
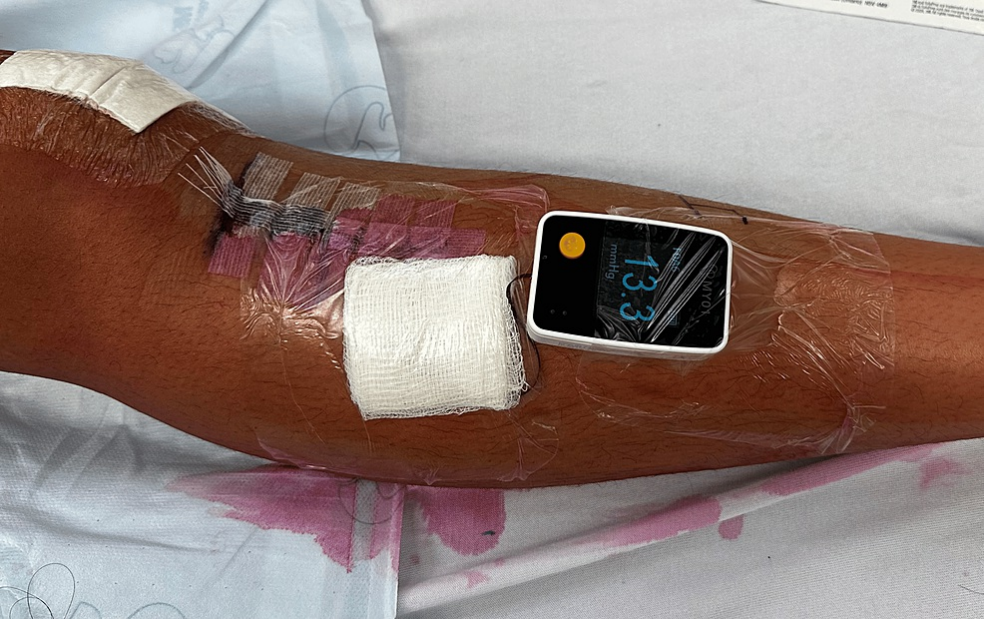
MY01 (MY01 Inc.) device after insertion in the leg. The device was laced in the anterior compartment as per protocol, allowing the monitoring of continuous pressure trends in all four compartments.

A MEMS-based pressure monitor [6,7] was inserted as per protocol in the anterior compartment of the operated leg (Figure 3).

This device produces a graphic output sent wirelessly to a phone app as well as presenting a discrete pressure output on a display screen on the leg itself for direct pressure observation. The device allows pressure trend measurement and calculation of perfusion pressure. Radiographs were obtained at this time, but there were no signs of hardware failure (Figures 1, 2). As shown in the figures, the sensor is placed in the muscle belly. Over an hour, the patient was observed under adequate sedation. Continuous pressure measurement showed a trending decrease in pressures with no dangerous values [2]. The decision not to perform a fasciotomy was made. The existing adductor canal block catheter was removed, and a new catheter was re-inserted under ultrasound guidance in the operating room.

Immediately after the operating room observation period, the patient had near-complete pain relief and was able to dorsiflex her feet and all toes with no increase in pain. She had no pain on palpation of the entire lower limb in which all muscle compartments were soft. The patient was provided an oral regimen of acetaminophen, ibuprofen, and minimal opioids along with the nerve block. The block was discontinued on postoperative day 2 and she was successfully discharged on oral pain medication.

## Discussion

Examination for CS is difficult even in alert and communicative patients. This case illustrates both the difficulties in examining and establishing an accurate treatment plan in a pediatric patient with an underlying neurodevelopmental disorder. Objective values in the form of continuous pressure readings are fortunately now obtainable. All previous diagnostic tools were only subjective. MEMS-based pressure sensors are a new tool with increased accuracy. They also allow placement of a sensor directly into the muscle belly [7], avoiding any artifacts with external sensors at the end of a water column. Both preclinical and clinical studies have shown that this objective feedback is more effective than previous technology [6-9]. There is real-time pressure monitoring in a continuous manner that had not previously been available. The recent studies centered on this process have established new standards in CS diagnosis and treatment [8,10,11]. The important innovation in care with continuous monitoring is avoiding unnecessary fasciotomies, with all the attendant complications, while allowing early recognition of true positives. Earlier work by Duckworth and McQueen [2] among others is validated with recent clinical data from Leighton et al. [6] and others on continuous compartment pressure monitoring. We know from the literature that clinical signs are not always reliable and that older technology for monitoring pressure is unreliable [1,7]. Newer technology as illustrated in this case aids in diagnosing CS.

This device allowed the physician team for this patient to have a more meaningful objective data stream that made them confident enough to decide on treatment. Initial clinical examination was indicative of a need for compartment release, an invasive surgery that leaves the muscle bellies open to the air for several days. This patient was having unremitting crescendo pain with increasing pain upon passive movement of the muscles in her leg. Unfortunately, this patient was not the optimal patient for either diagnosis by clinical signs or protracted wound care protocols. Despite being a currently unorthodox approach to the clinical problem, the care map chosen by the treating physicians allowed pain relief, diagnosis, and avoidance of fasciotomy. The concept of using a continuous pressure device in obtunded or otherwise unexaminable patients is supported by this case study.

## Conclusions

Continuous biomarker monitoring is standard in medicine. For example, we have already gone from intermittent measurements to continuous data in systemic blood pressure monitoring. Continuous monitoring allows better tracking of disease processes. New sensor technology has made continuous measurements readily available. MEMS-based continuous compartment pressure measurement represents a logical step in CS biomarker monitoring. It aids in diagnosing all patients at risk of missing CS due to the known unreliability of the clinical examination. This is particularly true in obtunded or unexaminable patients. Use of continuous biomarker monitoring has revealed high specificity and sensitivity, allowing early diagnosis and avoidance of false positives. Data regarding continuous compartment pressure monitoring are limited in the literature. However, it has been shown in some centers that continuous pressure in combination with physical examination decreases time to the operating room in CS patients. This new MEMS-based device allows remote monitoring of pressure measured inside the muscle itself. This particular device was used in this patient and permitted accurate diagnosis of risk for CS. It also allowed placement of a nerve block in this difficult-to-examine patient to decrease anxiety and allow continued assurance of no CS. Continuous pressure measurement plays an important role in the diagnosis of true-positive cases while importantly also decreasing the number of unnecessary surgeries. This represents a new process for controlling morbidity and mortality, as well as minimizing hospital costs and ensuring maximal outcomes.
